# Effect of Epoxy Structure on Properties of Waterborne Coatings and Electrical Steel Laminates

**DOI:** 10.3390/polym14081556

**Published:** 2022-04-11

**Authors:** Cornelia Marchfelder, Robert Pugstaller, Gernot M. Wallner, Oliver Brüggemann, Maëlenn Aufray

**Affiliations:** 1 Institute of Polymeric Materials and Testing & Christian Doppler Laboratory for Superimposed Mechanical-Environmental Ageing of Polymeric Hybrid Laminates (CDL-AgePol), Johannes Kepler University Linz, Altenbergerstraße 69, 4040 Linz, Austria; robert.pugstaller@jku.at (R.P.); gernot.wallner@jku.at (G.M.W.); 2 Institute of Polymer Chemistry, Johannes Kepler University Linz, Altenbergerstraße 69, 4040 Linz, Austria; oliver.brueggemann@jku.at; 3 CIRIMAT, Université de Toulouse, CNRS, INP-ENSIACE, T 4 allée Emile Monso- BP44362, CEDEX 4, 31030 Toulouse, France; maelenn.aufray@toulouse-inp.fr

**Keywords:** epoxy equivalent weight, waterborne epoxy, thin adhesive layer, electrical steel laminates, fatigue fracture mechanics

## Abstract

Epoxy varnishes are of high relevance to advanced steel laminates for the transformation of electric energy. Structure–property correlations of epoxy varnishes, coil coatings and electrical steel laminates are poorly described. Hence, the main objective of this paper was to develop, implement and evaluate well-defined waterborne model epoxy varnishes for electrical steel laminates, and to elucidate structure–property correlations. Adhesives with systematically varied equivalent epoxy weight (EEW) based on bisphenol-A-diglycidyl ether (DGEBA) were investigated and used to formulate waterborne varnishes. Crosslinking agent dicyandiamide (DICY) was added in an over-stoichiometric ratio. The waterborne model varnishes were prepared by shear emulsification at elevated temperatures. The model varnishes in the A-stage were applied to electrical steel using a doctoral blade. At a peak metal temperature of 210 °C, the coatings were cured to the partly crosslinked B-stage. Coated steel sheets were stacked, laminated and fully cured to C-stage at 180 °C for 2 h. For laminates with an epoxy adhesive layer in the C-stage, glass transition temperatures (T_G_) in the range of 81 to 102 °C were obtained by dynamic mechanical analysis in torsional mode. Within the investigated EEW range, a negative linear correlation of EEW and T_G_ was ascertained. Presumably, higher EEW of the varnish is associated with a less densely crosslinked network in the fully cured state. Roll peel testing of laminates at ambient and elevated temperatures up to 140 °C confirmed the effect of EEW. However, no clear correlation of roll peel strength and glass transition temperature was discernible. In contrast, fatigue fracture mechanics investigations revealed that hydroxyl functionality and crosslinking density were affecting the crack growth resistance of laminates in a contrary manner. The energy-based fracture mechanics approach was much more sensitive than monotonic peel testing.

## 1. Introduction

Electrical steel laminates based on epoxy adhesive layers are of high relevance for the transformation of electric energy [1,2,3,4]. By the stacking and bonding of thin electrical steel sheets, eddy current losses are reduced and the efficiency of electric machines is raised [3]. Epoxy plies act as multifunctional separation layers providing electrical isolation and full-surface bonding. To maintain the high power density of electric engines or generators, the adhesive layer thickness should be uniform and restricted to a minimum. Lamination and isolation of electrical steel with epoxy-based adhesives offers many advantages, but also a few drawbacks. Compared to classical joining methods like welding or interlocking, the high temperature performance of adhesively bonded laminates is limited [3].

Epoxy adhesives and coatings provide remarkable properties such as good adherence to various substrates, thermal stability or electrical isolation. Waterborne epoxy varnishes, especially, allow for multifunctional coatings with a thickness in the µm range. Therefore, low viscosity varnishes are required. Both solvent-based and waterborne systems have been developed. Due to environmental and legislative restrictions, waterborne epoxy varnishes are preferred. Further advantages of waterborne epoxy varnishes are ease of applicability by coil coating processes, low volatile organic content (VOC) and odor or reduced flammability [5,6,7]. In order to enhance interfacial adherence, hydroxyl groups, allowing for bonds to the oxidic passivation layer of the electrical steel substrate, are essential. While secondary bonds, such as hydrogen and van der Waals forces, provide good adherence at ambient conditions, chemical covalent or coordinative bonds are of special relevance for long-term behavior under hot-humid conditions [8,9,10,11].

Liquid epoxy varnishes (A-stage) and coatings are applied in a two-stage process. First, the electrical steel substrate is coated with the waterborne epoxy varnish system, which is then dried and partly cured to the B-stage. The coated electrical steel substrates can be stored for up to several months. In a second step, the sheets are cut to the desired geometry and stacked. By heat press lamination, a high curing degree of the epoxy adhesive (in C-stage) is ensured. Processing temperature, time and pressure are dependent on the epoxy formulation but also on the geometry of the laminate [2,12,13].

Maximum service temperatures of electrical steel laminates are around 180 °C [14]. The glass transition temperature of commonly used epoxy adhesive systems is lower. Hence, the crosslinking density of epoxy networks, which is usually proportional to the mechanical properties, is of utmost importance [15,16,17]. In addition to the bulk properties of adhesives, interfacial bonding to the passivation layer of the substrate is essential. In a few studies, structure–property correlations between the epoxy equivalent weight (or hydroxyl group concentration) of non-waterborne epoxies and adhesive strength at ambient temperatures have been established [8,10]. A higher EEW value, associated with increased monomer chain length, results in a reduced crosslinking density and enhanced hydroxyl group concentration [18]. So far in the literature, no specific attention has been given to the structure–property correlations of waterborne epoxy varnishes allowing for micrometer thick adhesive layers in electrical steel laminates.

Hence, the main objective of this paper was to investigate the effect of structural parameters of waterborne epoxy varnishes on the thermomechanical and mechanical properties of electrical steel laminates. Varnish systems with varying EEW values were formulated and applied. Thermomechanical analysis was performed to determine the glass transition. The adherence strength of laminates was characterized by roll peel testing at ambient and elevated temperatures. Furthermore, fatigue crack growth kinetics were examined at ambient conditions.

## 2. Materials and Methods

### 2.1. Materials

**Figure 1:**
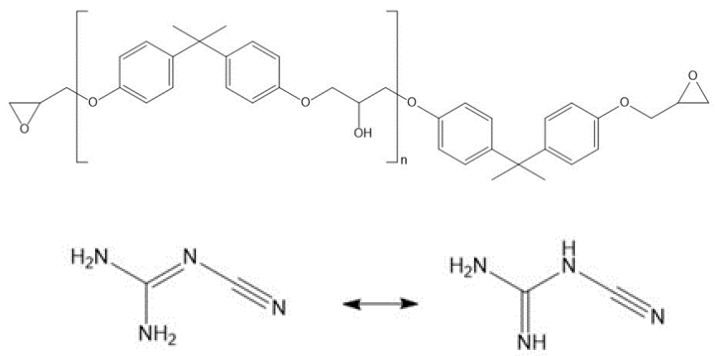
Chemical structure of DGEBA with repeating unit n (**top**) and DICY (**bottom**) with its two tautomeric forms, diamino (**left**) and amino-imino (**right**).

The investigated epoxy adhesives were based on bisphenol-A-diglycidyl ether (DGEBA). Three different types of epoxides (Table 1) with varying repeating units n (Figure 1) were used. Adhesives were classified by their epoxide equivalent weight (EEW) depending on their repeating unit n. The EEW values ranged from 475 to 1900 g/mol. The letter “A” indicates Araldite products supplied by Huntsman International LLC (Salt Lake City, UT, USA). A-1800 represents Araldite GT 6097. In contrast, “D” indicates products from the D.E.R. series manufactured by Olin Corporation (Clayton, MO, USA). D-900 was delivered as D.E.R. 664UE and D-500 as D.E.R. 671. Epoxides with an EEW value of more than 400 g/mol are solid. The melting temperature range (T_M_) according to the data sheets is listed in Table 1.

Due to its latent hardening behavior, the curing agent dicyandiamide (DICY) was selected. A solid micronized DICY powder, DYHARD 100SH (Alzchem, Trostberg, Germany), with a maximum particle size of 10 µm was used. According to the data sheet, the melting point was in the range from 209 to 212 °C. DICY reveals an amino-imino or a diamino form (see Figure 1).

### 2.2. Preparation of Varnishes, Coatings and Laminates

**Figure 2:**
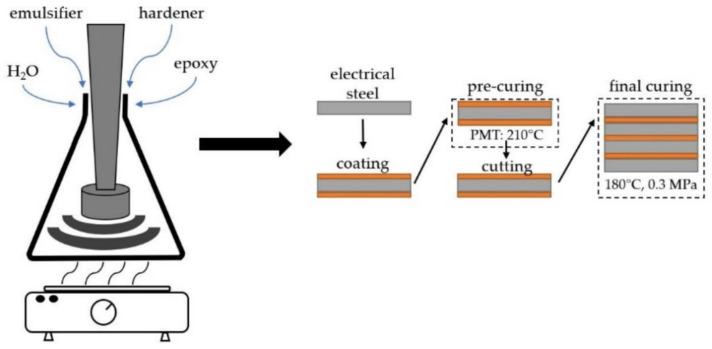
Schematic illustration of varnish preparation (**left**) and electrical steel coating, stacking and lamination (**right**).

The water content of the varnish formulations was fixed to 45 m% (of the total system). Emulsifier Poloxamer 407 (Croda, Snaith, UK) was used. According to the formulation guideline of the supplier, the emulsifier was 10 m% related to the epoxy base resin. Depending on the EEW, two different formulation approaches were employed (Figure 2 left). For solid epoxies with an EEW value up to 600 g/mol, homogenization of the base resin and the emulsifier was possible by stirring at temperatures well below the boiling point of water (~85 °C). After homogenization, distilled water was added dropwise at an elevated temperature under constant agitation. As such, a dropping funnel was used. Epoxies with EEW values of more than 600 g/mol were dissolved in acetone prior to addition of the emulsifier. This allowed for homogenization at temperatures below the boiling point of water. The emulsions were prepared using an DMX 160 emulsifying shear head (Dynamic, Kehl, Germany). Hence, axial and radial flow forces were ensured for optimized emulsification. For higher molecular weight systems, acetone was evaporated after emulsification. As the waterborne epoxy systems A-1800, D-900 and D-500 were prone to foaming, 0.75 m% of the defoaming agent BYK-016 (BYK, Wesel, Germany) was added.

The amount of curing agent DICY was calculated dependent on the amine H equivalent weight (eq wt), as well as the epoxy equivalent weight. The amine H eq wt value of DICY was assumed to be 21 based on 4 reactive sites [19]. As the epoxy equivalent weight, the lowest value in the given EEW range was considered. DICY content, in parts per hundred (PHR), was calculated according to:

(1) $$\mathrm{PHR}\;\left(\mathrm{curing}\;\mathrm{agents}\right)=\frac{\mathrm{amine}\;H\;\mathrm{eq}\;\mathrm{wt}}{\mathrm{epoxy}\;\mathrm{eq}\;\mathrm{wt}}\times 100$$

An over-stoichiometric amount of the curing agent was added to an excess of 1.1 to 1 (DICY to epoxy) to ensure full crosslinking, as topological restrictions may limit the maximum achievable conversion [20]. DICY was dissolved in methoxy propanol to avoid agglomeration.

Using a suitable doctoral blade, the model varnishes were applied on M800-50A grade electrical steel sheets (according to DIN EN 10106) with a thickness of 0.5 mm. The coated steel sheets were pre-crosslinked at a peak metal temperature (PMT) of 210 °C (see Figure 2, right). Both sides of the electrical steel sheets were subsequently coated. The coating on the top side of the steel sheet was dried for 10 s at 210 °C. Then, the bottom side was coated. Afterwards, the electric steel sheet with coatings on both surfaces was exposed in an oven at PMT of 210 °C for 25 s. In this way, partly cured coatings in B-stage could be applied to both sides. Subsequently, the coated sheets were cut to 200 × 25 mm^2^ stripes for roll peel testing and 150 × 25 mm^2^ for fatigue crack growth characterization. Laminates consisting of 4 steel layers (roll peel specimen) and 6 steel plies (fatigue crack growth specimen) were stacked. The laminates were fully crosslinked in a convection oven at a temperature of 180 °C for 2 h with 0.3 MPa pressure applied.

### 2.3. Characterization of Raw Materials, Coatings and Laminates

The structure of the epoxides and the emulsifier was assessed by infrared spectroscopy. A Spectrum 100 FTIR (Perkin Elmer, Waltham, MA, USA) in ATR-mode with a MCT detector was used. The wavenumber range from 4000 to 600 cm^−1^ was evaluated with 16 scans and a resolution of 4 cm^−1^. The applicability of the varnish was examined after coating by optical microscopy. Therefore, a VHX 7000 (Keyence, Mechelen, Belgium) digital microscope was employed. Images were recorded using the VH-ZST objective at a magnification of 20×.

**Figure 3:**
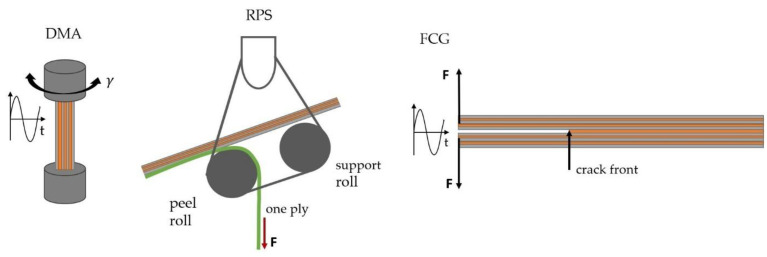
Schematic configurations of test setups for dynamic mechanical analysis (DMA, **left**), monotonic roll peel strength testing (RPS, **middle**) and fatigue crack growth assessment (FCG) using a double cantilever beam (DCB) specimen (**right**).

The thermomechanical behavior of the laminates was characterized by Dynamic Mechanical Analysis (DMA) according to DIN EN ISO 6721 on the rheometer Physica MCR 502 (Anton Paar, Graz, Austria) in torsional mode. The specimen size was 50 × 10 × 2 mm^3^. DMA experiments were performed in a temperature range from 30 to 220 °C with a heating rate of 3 K/min and a strain amplitude of 0.1%. The frequency was set to 1 Hz. Storage modulus and loss factor were evaluated as functions of temperature. The glass transition temperature was deduced from the maximum of the loss factor. A schematic drawing of the DMA test setup is depicted in Figure 3 (left).

Adhesive strength was determined by roll peel testing on a universal testing machine Z020 (ZwickRoell, Ulm, Germany) according to EN 1464. A schematic roll peel test configuration is illustrated in Figure 3 (middle). The specimen size was 200 × 25 mm^2^ consisting of 4 electrical steel layers. One ply of the laminate was peeled off from the remaining plies. The test rate was 100 mm/min. Specimens were tested at ambient conditions (23 °C, 50%rh) as well as 100 and 140 °C in a temperature chamber. At ambient conditions, three repetitions for each system were carried out, while at 100 °C and 140 °C the number of repetitions was set to two. Roll peel strength was deduced by averaging the force normalized by specimen width over the displacement, cutting off the first and last 25 mm of displacement.

Fatigue crack growth behavior was characterized by an electro dynamic machine ElectroPlus E3000 (Instron, Norwood, MA, USA) according to ISO 15024. Therefore, double cantilever beam (DCB) specimens were used. A schematic DCB test configuration is depicted in Figure 3 (right). Displacement controlled measurements in mode I were carried out with a maximum amplitude of 3 mm. The sinusoidal displacement had a frequency of 5 Hz and a R-ratio (minimum to maximum displacement) of 0.1. A compliance-based method was used to determine the crack length as a function of number of cycles. The debonding energy or strain energy release rate was calculated considering a simple beam theory approach. Specific details are given in [3].

## 3. Results and Discussion

**Figure 4:**
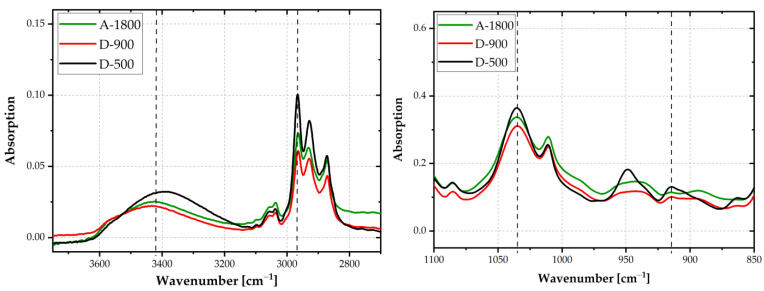
FTIR-ATR spectra in the range from 3700 to 2700 cm^−1^ (**left**) and 1100 to 850 cm^−1^ (**right**) of epoxies with average EEW values of 500, 900, and 1800 g/mol.

The chemical structure of the epoxy resins was evaluated by FTIR spectroscopy. IR-spectra are depicted in Figure 4. Depending on the molar mass of the epoxides, the oxirane ring intensity at 915 cm^−1^ varied. The lower the number of repeating units (n), the more pronounced was the oxirane ring peak. The oxirane ring functionality of a DGEBA molecule is equal to two. The intensity of this peak leveled off at EEW values of 900 and 1800 g/mol. Hence, the highest intensity of the oxirane ring was detected for epoxy D-500, with a decrease in intensity towards the highest EEW adhesive A-1800. By weighting the oxirane peak at 915 cm^−1^ with the C-O-C ether stretching peak at 1035 cm^−1^ [21] (vertical dashed lines), this trend was confirmed. The ratio was 0.36 for D-500 and 0.33 for D-900 and A-1800.

Due to water uptake at the surface, no clear trend as to the OH-stretching peak at 3400 cm^−1^ was discernible. With increasing n, the intensity of the OH-absorption peak should become more pronounced. By weighting the peak at 3400 cm^−1^ with the CH_3_ stretching peak at 2965 cm^−1^ (vertical dashed lines), a clear indication for higher amounts of OH-groups was ascertained for A-1800 or D-900, compared to D-500. The peak ratios were 0.36 for A-1800 and D-900, and 0.32 for D-500. These findings are in good agreement with data in the literature [22,23].

**Figure 5:**
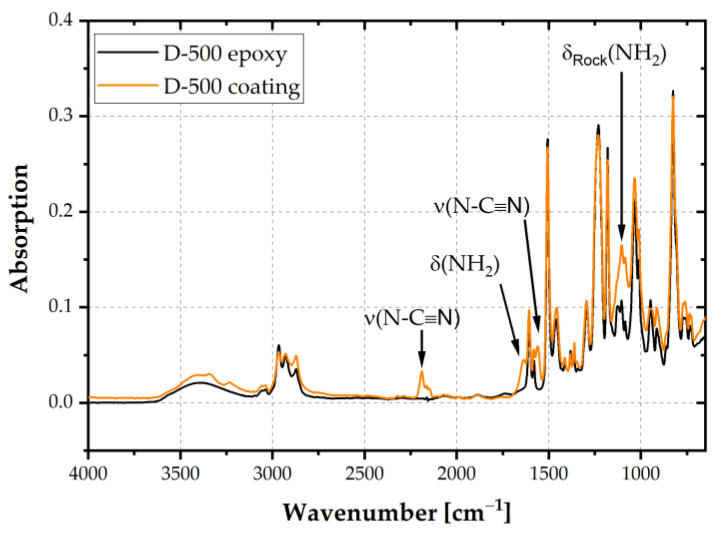
FTIR-ATR spectra of the epoxy resin D-500 and the coating in B-stage.

The formulated waterborne epoxy varnishes were applied to electrical steel, dried and pre-cured to coatings in B-stage in a convection oven at a peak metal temperature of 210 °C. The coating thickness in B-stage was 5 µm for all epoxy thermosets. Figure 5 shows an exemplary comparison of the epoxy resin D-500 with the coating in B-stage. The spectra were normalized to the peak at 1509 cm^−1^ as the intensity of the phenylene stretching vibration did not change during crosslinking. Peaks relevant to the curing agent DICY were found at 1638 cm^−1^ and 1100 cm^−1^ due to resonant states of the bending vibration of NH_2_. The oligomer formation of DGEBA and DICY was proven by asymmetric stretching peaks of N-C≡N at 2187 cm^−1^, which is characteristic for linear oligomers. Furthermore, the peak at 1560 cm^−1^ (ν (N-C≡N)) was attributed to the guanidine unit resulting from oligomers [23]. The peaks marked in Figure 5 are characteristic for DICY and oligomers with DGEBA. Hence, partial crosslinking of the investigated coatings was confirmed.

**Figure 6:**
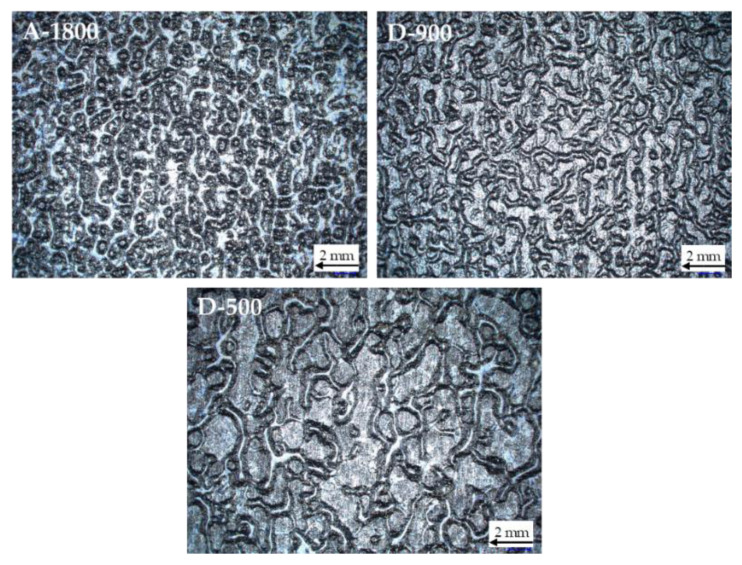
Topographic images of B-stage coated electrical steel sheets based on the epoxy adhesives A-1800, D-900 and D-500.

The topography of the applied coatings in B-stage is depicted in Figure 6. Full surface wetting was ascertained optically and confirmed by FTIR-spectroscopy for all investigated varnish formulations. However, most of the coatings exhibited thickness variations and a wavelike topography. The wavelength of the pattern was finer for coatings based on epoxy adhesives of higher EEW. Most likely, the waviness is dependent on the viscosity of the varnish. This effect is described in the literature as orange peel. A main reason for orange peel effects is non-optimized solvent content of the varnish system [24].

**Figure 7:**
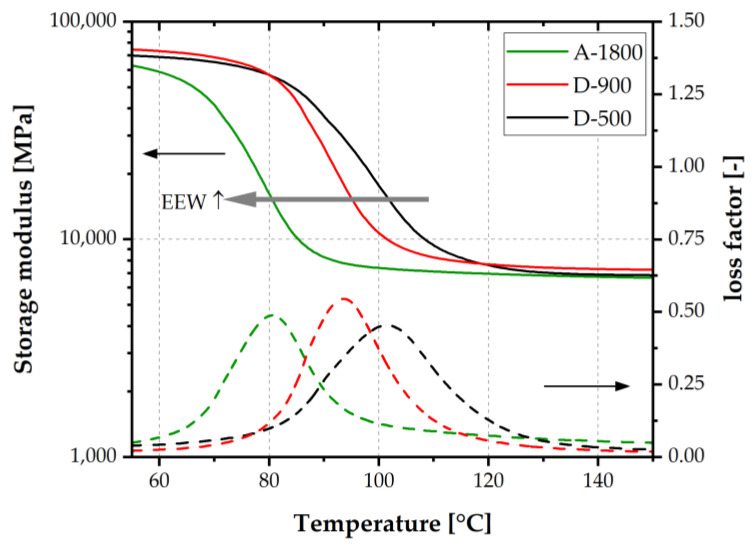
Storage modulus and loss factor curves of electrical steel laminates adhesively bonded with epoxy varnishes with EEW values of 500, 900 and 1800 g/mol.

Storage modulus and loss factor thermograms of the investigated laminates are depicted in Figure 7. While storage modulus of the laminate was about 70 GPa in the glassy state below glass transition temperature of the adhesive, it dropped by one magnitude to 7.3 GPa above T_G_ in the rubbery state of the adhesive. The T_G_ value was deduced from the maximum of the loss factor. The highest value of 102 °C was obtained for the laminate based on varnish D-500. In contrast, lower T_G_ values of 94 °C and 81 °C were detected for laminates with coatings based on higher molar mass epoxies D-900 and A-1800, respectively. Hence, in agreement with findings in the literature [16], glass transition temperature was indirectly proportional to the EEW of the epoxy thermoset. This relationship was attributed to a lower crosslinking density of high EEW adhesives. The theoretical crosslinking density for adhesive A-1800 was a factor of 3 lower than that of D-500. The absolute values of the storage modulus in the glassy and rubbery plateaus did not reflect the pronounced effect of EEW. The storage modulus was mainly affected by the electrical steel substrate. Due to an adhesive layer thickness well below 10 µm, small fluctuations in thickness had a strong impact on scattering of modulus values, especially in the rubbery state. For laminates based on commercially available varnishes, higher glass transition temperatures, in the range from 110 to 117 °C, were reported in [25], slightly dependent on the electrical steel grade. As described by Aufray and Roche [26], chemical interactions of amine-based curing agents and metal oxide passive layers could also have an impact on glass transition temperature values and the macromolecular structure of the thermoset at the interphase.

The width of the loss factor peak at 50% of the maximum value was highest for laminates based on D-500, and lowest for D-900. In agreement with the findings of Misra [16], the crosslinking network of coatings made from adhesives of lower EEW values was, presumably, less perfectly associated with wider loss factor peaks.

**Figure 8:**
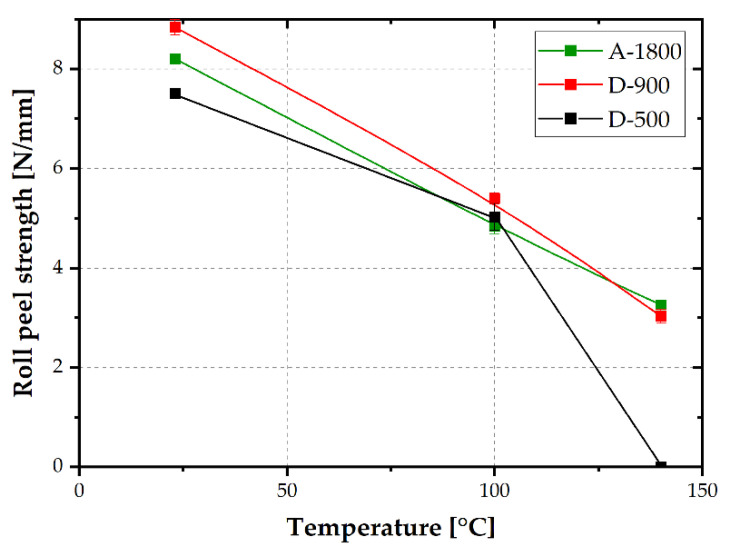
Roll peel strength at 23, 100 and 140 °C of laminates made from epoxy varnishes with EEW values of 500, 900 and 1800 g/mol.

Adherence strength was determined by roll peel testing at 23, 100 and 140 °C. The temperature-dependent roll peel strength values of the investigated laminates with adhesives of differing EEW values are plotted in Figure 8. At ambient temperature, the lowest roll peel strength, of 7.5 N/mm, was determined for D-500. Adhesives of higher EEW (A-1800 and D-900) exhibited increased roll peel strength, higher than 8 N/mm. Presumably, a lower crosslinking density is beneficial for roll peel strength at ambient conditions. Slightly lower crosslinking density would allow for less brittle fracture and, therefore, higher roll peel strength. A similar trend was proven by Pugstaller et al. [25] for laminates made from a commercially available varnish. The roll peel strength values were in a similar range of about 8 N/mm.

At a test temperature of 100 °C, which was within the glass transition of the investigated adhesives, a pronounced decrease in roll peel strength was ascertained. This effect could be attributed to an enhanced segmental mobility of the polymeric adhesive at increasing temperatures [27]. The drop was less pronounced for laminates based on D-500, with higher storage modulus values at 100 °C due to a slightly higher glass transition temperature. At 140 °C, well above T_G_, a further drop of roll peel strength, to about 3 N/mm, was discernible. The laminate based on D-500, with the highest T_G_, showed inferior adherence at 140 °C and could not be tested. Better adherence performance at 140 °C was obtained for thermosets with higher OH functionality (i.e., D-900 and A-1800). By comparing T_G_ or OH functionality with roll peel strength at 100 °C or 140 °C, a much higher impact of OH functionality was deduced. Hence, OH functionality is presumably much more important than crosslinking density. Due to hydroxyl groups, different bonds such as secondary or covalent bonds to the metal oxide surface are established [8,10,28,29].

As confirmed by IR spectroscopy in reflection mode using an Ulbricht sphere, the fractured surfaces of the investigated laminates, roll peel tested at 23, 100 and 140 °C, revealed, predominately, interfacial failure. This supports the hypothesis of high relevance of OH functionality for improvement of interfacial debonding performance.

**Figure 9:**
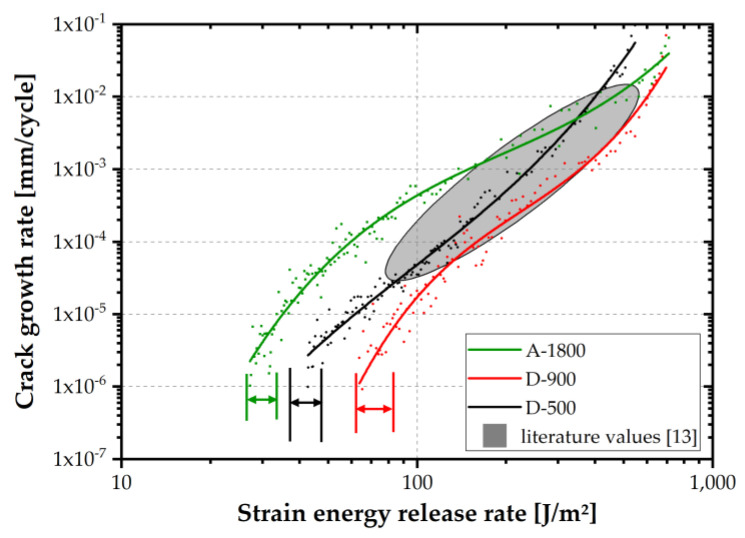
Crack kinetic curves at ambient temperature of laminates made from epoxy varnishes with EEW values of 500, 900 and 1800 g/mol compared to values from the literature [13], marked in grey.

In order to test adherence performance under more service-relevant conditions, fatigue fracture mechanics tests were performed. In roll peel testing, most of the peeling energy is attributable to plastic deformation of the substrate [30]. This effect could be excluded by fracture mechanics delamination testing using a double cantilever beam specimen (DCB). In Figure 9, crack growth kinetics curves of the laminates tested at ambient temperature are depicted. The curves can be divided into three different regions: the threshold, stable crack growth and unstable crack growth regime. In the threshold regime (ultra-low crack growth rates), the adhesive based on A-1800 showed lower strain energy release rate threshold values (G_th_) than laminates prepared from D-900 or D-500 epoxies. Presumably, slow crack propagation in the threshold regime of laminates based on epoxies of low and intermediate EEW values was less pronounced due to better adherence to the passive layer of the substrate. Additionally, at low strain energy release rate values, the plastic zone size at the crack tip is less- or non-constrained by the substrate. If the plastic zone is constrained, the crack tends to propagate within the adhesive layer, associated with failure in cohesive manner [13]. Therefore, at slow crack propagation rates, better cohesive strength of the epoxy layer would result in enhanced crack growth resistance.

In the stable crack growth regime (II), the lowest crack propagation rate was obtained for laminates based on D-900. Compared to fatigue crack growth data for laminates based on commercially available varnish systems [13] (see grey area in Figure 9), a similar fatigue crack growth resistance was confirmed in regime II. For monotonic fracture mechanic tests with a high crack tip radius, a trend of maximum fracture toughness at intermediate EEW values was also reported by Levita et al. [31]. Furthermore, for amorphous polymeric materials, a higher crosslinking density was related to increased yield stress and reduced strain at break [32]. These two contrary effects necessitate an optimization and a compromise between crosslinking density and ductility. A better stress distribution at the crack tip could be achieved by higher inner mobility (e.g., at higher temperatures). Nevertheless, a certain level of yield stress has to be exceeded. In addition to bulk adhesive properties, the plastic zone at the crack tip is constrained at higher strain energy release rates by the substrate. Subsequently, the crack tends to propagate in the substrate-adhesive interface. Under such conditions, substrate-adhesive interactions (eg., OH bonds or covalent bonds) are of utmost relevance to ensure sufficient fatigue crack growth resistance [13].

The behavior of high EEW laminates (i.e., A-1800) was slightly better in the unstable crack growth regime. Interestingly, the ranking of roll peel strength values and crack growth resistance was equivalent in the unstable crack growth regime. However, contrary to roll peel testing, the DCB specimen failed, mainly in a cohesive manner. This difference is most likely related to excessive plastic deformation of the substrate during roll peel testing.

**Figure 10:**
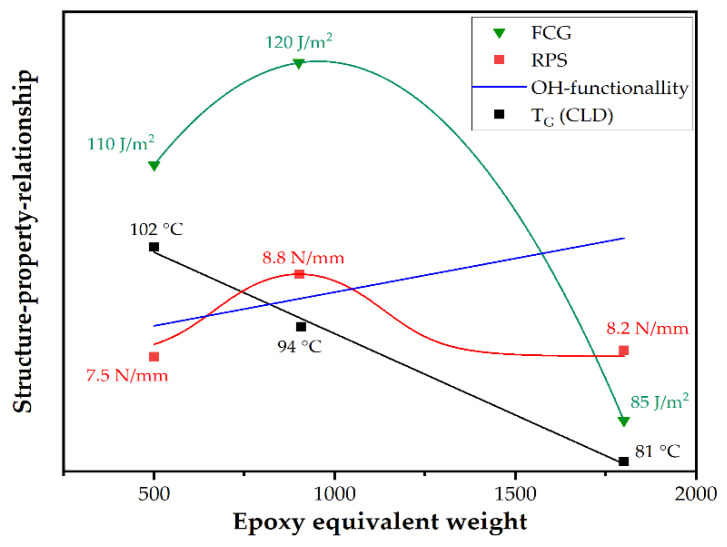
Schematic relationship of OH functionality, crosslinking density (CLD), roll peel strength (RPS) and fatigue crack growth kinetics (FCG) at ambient temperature and the epoxy equivalent weight (EEW) of the adhesive in electrical steel laminates.

In Figure 10, structure–property relationships, of molar mass or epoxy equivalent weight and the investigated properties, are depicted schematically. As confirmed by DMA, raising EEW causes a decrease in crosslinking density (CLD). In contrast, the hydroxyl functionality affecting the adherence to the substrate raises with higher molar mass of the epoxy resin. Both roll peel strength (RPS) and fatigue crack growth kinetics (FCG) at ambient temperature peak at intermediate EEW values of 900 g/mol. At low or high EEW values, roll peel strength and fatigue crack growth kinetics are lower, due to poor adherence (at low EEW) or less crosslinking density (at high EEW). However, the effect of hydroxyl groups on the epoxy-metal adherence is still under discussion [33,34,35,36]. The postulated schematic relationships do not account for chemical interactions of amine-based hardeners and metal substrates [26], which have been investigated and ascertained in a recent study [37]. Future research will focus on the potential effects of interfacial interactions and interphase formation in the coating and lamination process on the adherence performance of steel/epoxy laminates.

## 4. Conclusions

Waterborne varnishes for electrical steel laminates based on bisphenol-A-diglycidiyl ether (DGEBA) resins with varying epoxy equivalent weights (EEW) were prepared and investigated. The EEW values of the epoxy resins ranged from 500 to 1800 g/mol. 10 m% of poloxamer emulsifier was added to the formulation. As curing agent, dicyandiamide (DICY) was used in an over-stoichiometric amount. Electrical steel sheets were coated with the investigated varnish systems. After pre-curing, the coated steel sheets were stacked and hot press-cured. The investigated laminates consisted of 4 or 6 electrical steel plies (roll peel or fatigue crack growth tests) and epoxy adhesive layers with a thickness of about 5 µm.

By infrared spectroscopy, a higher amount of oxirane ring groups, but a lower OH functionality, was confirmed for lower molar mass epoxy. The orange peel effect of partly cured coatings on electrical steel substrates was also dependent on the epoxy equivalent weight (EEW) of the adhesives. For higher EEW values of 1800 g/mol, a much finer orange peel structure was discernible, most likely attributable to a higher viscosity in the coating process.

The thermomechanical properties of laminates with fully cured adhesive layers was examined by DMA. The lowest glass transition temperature was obtained at 81 °C for the highest EEW adhesive, with 1800 g/mol. With decreasing EEW value, the glass transition values rose up to 102 °C for the adhesive based on an EEW of 500 g/mol. Hence, crosslinking density was correlated in a negative manner with the EEW value of the adhesive.

The adhesive strength of the laminates was assessed by roll peel testing at 23, 100 and 140 °C. Laminates based on adhesives of higher EEW values exhibited better adherence performance below, and above, glass transition. A maximum strength of about 9 N/mm at 23 °C was obtained, which was in good agreement with data from the literature [3]. At 100 and 140 °C, roll peel strength dropped by 50 and 75%, respectively. This drop was attributed to enhanced main chain mobility within or above the glass transition regime of the epoxy adhesives. Especially at 140 °C, the laminates based on the adhesive of lowest EEW failed prior to testing. In contrast, laminates with coatings of intermediate or high EEW value revealed roll peel strength values of 3 N/mm. Hence, it was clearly deduced that higher EEW, associated with more OH functional groups, is of utmost importance to mechanical strength at temperatures around glass transition or higher. Most likely, the OH functionality and associated interfacial bonds are of higher relevance than crosslinking density. Due to mainly interfacial failure of the investigated laminates at testing temperatures below and above glass transition, there is significant potential for further improvement of the varnish formulations. Special attention should be given to adhesion promoters or adequate surface treatment procedures.

Fatigue crack growth resistance was characterized at ambient conditions. The best performance in the threshold and stable crack growth regime was deduced for laminates based on the adhesive of intermediate EEW of 900 g/mol. At slow crack propagation rates, the plastic zone size was less constrained by the substrate. Therefore, higher cohesive strength of the epoxy layer resulted in increased crack growth resistance. In the stable crack growth regime, the higher crosslinking density was related to enhanced yield stress and reduced strain at break. Overall, it was deduced that a well-balanced ratio of crosslinking density and OH functionality is of high relevance to ensure good performance under near-service conditions.

Finally, structure–property correlations were established and deduced for waterborne epoxy varnish systems with systematically varied EEW values and electrical steel laminates. It was clearly shown that enhancement of glass transition temperature and associated crosslinking density did not necessarily lead to better mechanical performance of the laminates. Especially at elevated temperatures and under fatigue loading conditions, the adherence was dependent on both cohesive strength of the epoxy layer and interfacial bonding to the metal oxide passivation layer of the substrate. Hence, a well-balanced compromise of inner mobility and ductility of the epoxy layer and interfacial bonding to the substrate is essential for the optimization of monotonic and fatigue delamination resistance. The model varnish-based approach allowed for a clear separation of opposing or reinforcing effects. The investigations confirmed that the energy-based fatigue testing approach was much more sensitive for elucidation of material structure effects than conventional force-based, monotonic testing.

To also assess the effect of ageing on the adherence of electrical steel laminates, future research will focus on fatigue testing under superimposed mechanical stresses and environmental influences. Furthermore, potential interactions of the micrometer-thin epoxy coating with the passive layer of electrical steel substrates, and their consequences on the thermoset network structure, will be investigated.

## Figures and Tables

**Table 1:** Used epoxy base resins along with the designation, the supplier, the EEW value range and the estimated melting temperature (T_M_).

Designation	Supplier	EEW-Range, g/mol	Melting Temp., °C
A-1800	A	1695 to 1885	125 to 135
D-900	D	860 to 930	100 to 110
D-500	D	475 to 550	75 to 85

## Data Availability

Not applicable.
